# The complete mitochondrial genome of the hybrid of *Hoplobatrachus chinensis* (♀)×*H. rugulosus* (♂) and its phylogeny

**DOI:** 10.1080/23802359.2018.1450661

**Published:** 2018-03-14

**Authors:** Yu-Ting Cai, Lin Ma, Chen-Jie Xu, Peng Li, Jia-Yong Zhang, Kenneth B. Storey, Dan-Na Yu

**Affiliations:** a College of Chemistry and Life Science, Zhejiang Normal University, Jinhua, Zhejiang Province, China;; b College of Life Science, Nanjing Normal University, Nanjing, Jiangsu Province, China;; c Key Lab of Wildlife Biotechnology, Conservation and Utilization of Zhejiang Province, Zhejiang Normal University, Jinhua, Zhejiang Province, China;; d Department of Biology, Carleton University, Ottawa, Canada

**Keywords:** *Hoplobatrachus chinensis*, *H. rugulosus*, hybrid tiger frog, mitochondrial genome, phylogeny

## Abstract

The complete mitochondrial genome sequence of the hybrid of *Hoplobatrachus chinensis* (♀) × *H. rugulosus* (♂) was obtained in this study. The circular mitochondrial genome was 20,282 bp in length (including extra ND5 genes). Compared with the complete mitogenome of the parents, the results indicated that the mitochondria of the hybrid tiger frog was consistent with a maternal inheritance. Phylogenetic analyses using concatenated nucleotide sequences of the 11 protein-coding genes with two different methods (maximum likelihood and MrBayes analysis) both highly supported a close relationship of the hybrid frogs with the Chinese tiger frog (=*H. chinensis*).

The numbers of Chinese tiger frogs, *Hoplobatrachus chinensis*, are now listed in Appendix II of CITES in China. Although many Chinese researchers think that the Chinese tiger frog is correctly designated as *H. chinensis*, some researchers still believe it to be a synonym of *H. rugulosus* (Frost 2016). Alam et al. (2008), Pansook et al. (2012), and Yu et al. (2015) found that cryptic species existed in the Thailand tiger frog. To evaluate the viable hybrid, we sequenced the complete mitogenome sequence of the *H. chinensis* (♀) × *H. rugulosus* (♂) hybrid. The female of *H. chinensis* was from Jinhua (29°21′39″, 119°57′58″), Zhejiang, China whereas the male *H. rugulosus* was from the Huwawa farm of Jinhua, Zhejiang, China, originally imported from Thailand. All the samples were stored at –70 °C in the college of Chemistry and Life Science, Zhejiang Normal University. Total genomic DNA stored in the Lab Yu was extracted from the tail of a hybrid tadpole using a standard proteinase K/SDS digest extraction method followed by phenol–chloroform isolation and ethanol precipitation (Sambrook 2001). We amplified the entire mitogenome of the hybrid by normal PCR and long-and-accurate PCR (LA-PCR) methods according to Yu et al. (2012, 2015). Sequences were checked and assembled using SeqMan (Lasergene version 5.0) (Burland 2000). The genomic sequence has been deposited in GenBank with an accession number MG770308.

The complete mitochondrial genome of the hybrid of *H. chinensis* (♀) × *H. rugulosus* (♂) was 20,282 bp in length and included an extra copy of *ND5* and an extra copy of *tRNA^Met^* genes. The two control regions had identical nucleotide sequences, except for an extra TT in 5′-CR2.

To confirm the phylogenetic relationships between the hybrid of *H. chinensis* (♀) × *H. rugulosus* (♂) and other tiger frogs, phylogenetic analyses in MrBayes (BI) and maximum likelihood (ML) analyses were performed on the concatenated nucleotide dataset of 10 PCGs excluding *ND5*, *ATP8*, and *ND6* genes. Mitogenomes for 25 species from Dicroglossidae were downloaded from GenBank (Liu et al. 2005; Ren et al. 2009; Zhang et al. 2009; Zhou et al. 2009; Alam et al. 2010; Chen et al. 2011; Yu et al. 2012; Shan et al. 2014; Yu et al. 2015; Chen, Zhai, Zhang, et al. 2015; Zhang, et al. 2018; Chen, Zhai, Zhu, et al. 2015; Jiang et al. 2016) and used to analyse the phylogenetic relationship of the hybrid using *Occidozyga martensii* (Li et al. 2014) as the outgroup. The tree topologies excluding the branch length produced by BI and ML analyses were equivalent (Figure 1). The hybrid is a sister clade of *H. rugulosus* (HM104684, Chinese tiger frog). The hybrid has a closer relationship and smaller genetic distance from its female parent (Chinese tiger frog) than its male parent (Thailand tiger frog), which can be explained by the maternal inheritance characteristics of the mitochondria.

**Figure 1.**
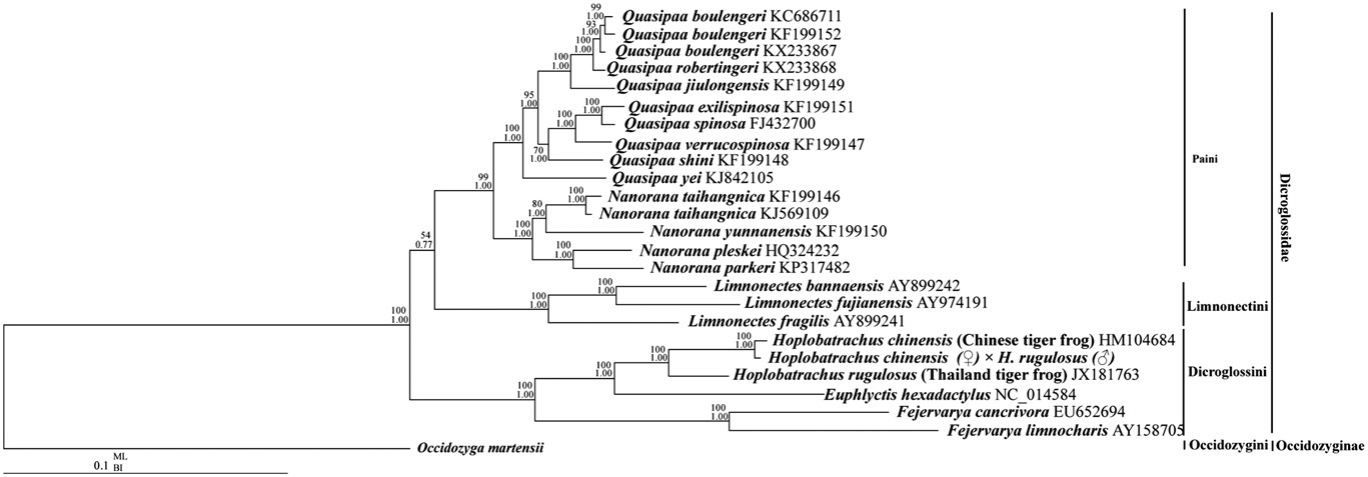
Phylogenetic relationships within Dicroglossidae based on 10 protein-coding genes using nucleotide datasets. Phylogenetic analyses using nucleotide datasets were carried out for the 25 dicroglossids based on all 10 protein-coding genes from their respective mt genomes. The tree was rooted with *Occidozyga martensii* as the out-group. Numbers above the nodes are the bootstrap values of ML on top and the posterior probabilities of BI on the bottom.
